# Host Subcellular Organelles: Targets of Viral Manipulation

**DOI:** 10.3390/ijms25031638

**Published:** 2024-01-29

**Authors:** Min Seok Song, Dong-Kun Lee, Chung-Young Lee, Sang-Cheol Park, Jinsung Yang

**Affiliations:** 1 Department of Physiology and Convergence Medical Science, Institute of Medical Science, College of Medicine, Gyeongsang National University, Jinju 52727, Republic of Korea; 2 Department of Microbiology, School of Medicine, Kyungpook National University, Daegu 41944, Republic of Korea; 3 Artificial Intelligence and Robotics Laboratory, Myongji Hospital, Goyang 10475, Republic of Korea; 4 Department of Biochemistry and Convergence Medical Science, Institute of Medical Science, College of Medicine, Gyeongsang National University, Jinju 52727, Republic of Korea

**Keywords:** virus, infection, organelles, endoplasmic reticulum, membrane, endocytosis, lysosome

## Abstract

Viruses have evolved sophisticated mechanisms to manipulate host cell processes and utilize intracellular organelles to facilitate their replication. These complex interactions between viruses and cellular organelles allow them to hijack the cellular machinery and impair homeostasis. Moreover, viral infection alters the cell membrane’s structure and composition and induces vesicle formation to facilitate intracellular trafficking of viral components. However, the research focus has predominantly been on the immune response elicited by viruses, often overlooking the significant alterations that viruses induce in cellular organelles. Gaining a deeper understanding of these virus-induced cellular changes is crucial for elucidating the full life cycle of viruses and developing potent antiviral therapies. Exploring virus-induced cellular changes could substantially improve our understanding of viral infection mechanisms.

## 1. Introduction

Throughout human history, viral infections have persistently threatened our well-being. Notably, viruses such as human immunodeficiency virus (HIV), influenza, and severe acute respiratory syndrome coronavirus 2 (SARS-CoV-2) have presented formidable challenges on a global scale [1,2,3]. In their quest for replication and survival, these viruses manipulate and exploit the cellular structures and processes of the host [4]. Viruses are intimately connected with their host cellular system, and they utilize the system. This system consists of an intricate molecular machinery and signaling pathways, which are employed to replicate their genome and produce new virions [5,6,7]. During virion assembly and release, some viruses use the membrane transport system to move viral particles between compartments and ultimately out of the cell [8,9]. Despite their apparent simplicity, viruses have evolved sophisticated strategies to steal the complex machinery of host cells.

Viruses encounter the extracellular matrix (ECM) as the first step of infection. The ECM, a complex non-cellular structure, is pivotal in maintaining the tissue architecture and modulating cell behavior [10,11]. Viral infections can profoundly influence the ECM, affecting its integrity and function through various mechanisms [12,13,14], such as activating inflammatory mediators [15]. Moreover, the interaction between viruses and the ECM is becoming more important to understanding viral pathogenesis and potential therapeutic interventions [16].

In addition to the ECM, viruses also target other cellular components, such as the cell membrane [17] and endocytosis mechanisms [18,19]. The cell membrane, with its myriad receptors, proteins, and lipids, is the primary gateway for viruses to enter and infect host cells [20,21,22]. Once inside, viruses can use endocytosis, a cellular mechanism that facilitates the internalization of extracellular molecules, to ensure their successful replication and spread [23]. The endoplasmic reticulum (ER) and mitochondria, vital organelles in eukaryotic cells, are not spared from viral manipulation. These organelles undergo significant structural and functional changes during viral infections, with viruses often repurposing them to create an environment conducive to their lifecycle [24,25,26].

Furthermore, the impact of viral infection in host cells extends to other cellular structures and processes, such as autophagosomes and lysosomes [27,28]. These organelles play crucial roles in cellular homeostasis, protein synthesis, and degradation [29,30]. Yet, viruses have evolved strategies to hijack these systems, using them to facilitate their replication, assembly, and release [31]. For instance, certain viruses induce the formation of biomolecular condensates, which serve as sites for viral RNA replication and nucleocapsid assembly [32,33]. Similarly, the lysosomal degradation system can be utilized by viruses for their exocytosis [34].

**Figure 1:**
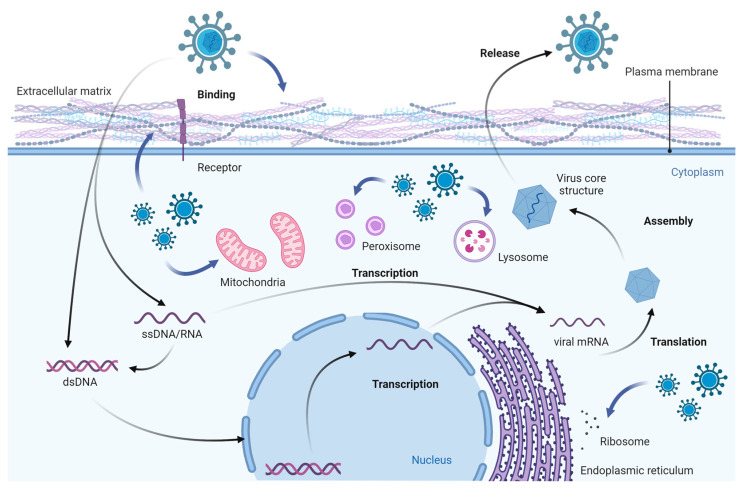
Viruses manipulate subcellular organelles to create conditions that facilitate their efficient proliferation. This figure was created with BioRender.com (accessed on 16 January 2024).

Since the onset of the COVID-19 pandemic, there has been a surge in virus research across various disciplines, leading to significant insights into the complex interplay between viruses and host cells and how viruses harness host cell mechanisms. Despite these advancements, a predominant tendency persists in virus research to interpret viral infections and their ensuing phenomena solely from the virus perspective. We believe that the intracellular changes induced by viral infections are not merely pathological occurrences; rather, they may be observed due to the activation of latent host cell functions or the reinforcement of their original capabilities. It is, therefore, imperative to also examine these changes from the host cell perspective (Figure 1). This review aims to provide a comprehensive examination of viral infections, specifically focusing on the morphological and functional changes of host subcellular organelles that occur during infections.

## 2. Viral Infection and Its Effects on Cell Components

### 2.1. Viral Infection Remodels the Extracellular Matrix

The ECM is a complex and dynamic non-cellular structure that plays a pivotal role in maintaining the tissue architecture and modulating cell behavior [35]. The ECM is not only crucial for the tissue architecture and cell behavior, but it also plays a significant role in modulating molecular and viral interactions within the tissue microenvironment [36,37]. Messenger proteins, including growth factors and cytokines, are sequestered within the ECM, which modulates their accessibility and function in cellular processes [38]. Additionally, ECM proteins, such as fibronectin and laminin, can serve as binding sites for certain viruses, thereby influencing viral attachment and subsequent infection [39,40,41]. In this context, understanding the interplay between the ECM and viruses can provide novel insights into viral pathogenesis and potential therapeutic interventions.

**Figure 2:**
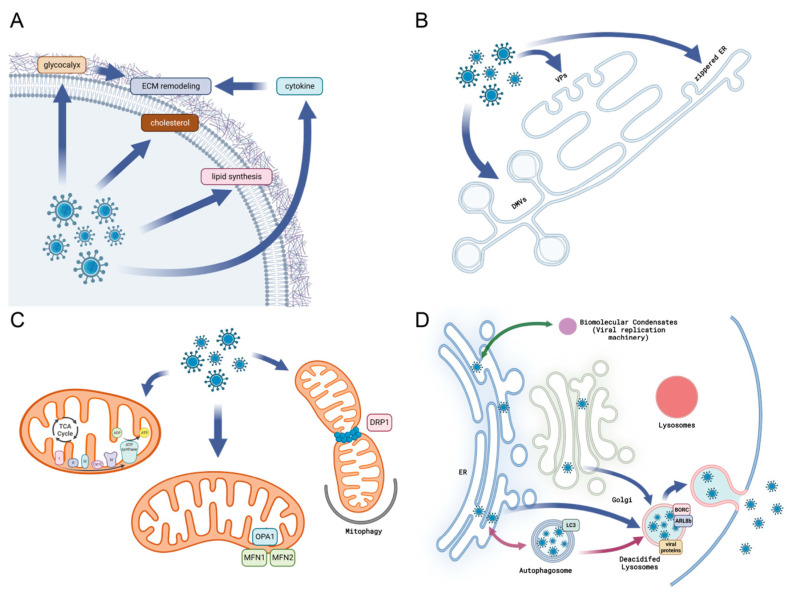
Viruses exploit the host cellular system to facilitate their replication and ensure their survival. (**A**) Viruses remodel the plasma membrane and extracellular by controlling their components. (**B**) Viruses induce the formation of membrane rearrangements including vesicle packets, double-membrane vesicles, or zippered ER within the host cell. (**C**) Viral components influence mitochondrial morphology and impact mitochondrial functions. (**D**) Viruses manipulate the formation of autophagosomes and biomolecular condensates to support their replication, and they exploit lysosomes for their eventual release. This figure was created with BioRender.com (accessed on 16 January 2024).

Viral infections exert profound effects on the ECM, influencing the activation of inflammatory mediators, including cytokines and chemokines, which are upregulated during viral infections (Figure 2A). For example, HCV infections have been shown to induce the expression of fibrogenic cytokines and ECM transcripts, highlighting the role of specific receptors in mediating fibrosis [42]. Furthermore, the interaction between tumor-supportive anti-inflammatory cytokines and microglia cells can activate distinct biological processes, including ECM remodeling, further underlining the relationship between inflammatory mediators and the ECM [43].

Chikungunya virus (CHIKV) infection induced an alteration in the ECM of the kidney and recellularized kidney scaffolds [44], although the precise mechanistic details remain elusive. An elevated level of IL-17 [45], which is a cytokine known to inflict damage on the ECM, was observed in CHIKV disease patients [46]. In rheumatoid arthritis (RA), IL-17 has been detected during the chronic phase of the disease, suggesting a potential link between CHIKV infection and ECM alteration [47,48,49]. Intriguingly, TNFAIP6, which plays a role in maintaining ECM stability and mediating cell migration with its hyaluronan-binding domain [50,51,52], exhibited a five-fold increase following CHIKV infection [53]. Notably, elevated TNFAIP6 levels have also been identified in patients with osteoarthritis and RA. Given these interrelated observations, a deeper exploration into the interplay between CHIKV infection and rheumatoid arthritis is needed.

Airway remodeling is a characteristic feature of asthma, and respiratory syncytial virus (RSV) infection has been implicated in its development [54]. RSV infection upregulates TLR4 expression and sensitizes epithelial cells to endotoxin [55]. RSV infection of human lung fibroblasts induces a hyaluronan (HA)-enriched ECM [56]. The virus infection alters the composition of the ECM by increasing HA production and the organization of it in higher-order structures. HA-enriched ECM binds mast cells and enhances their protease expression, which can contribute to inflammation [57]. RSV infection induces the HA-enriched ECM, potentially contributing to airway remodeling [56]. The RNA sequencing of airway cells from nasal swab samples revealed that the transcription levels of genes related to ECM increased specifically due to RSV infection [58].

Moreover, in the case of the highly pathogenic porcine reproductive and respiratory syndrome virus (HP-PRRSV) on porcine pulmonary microvascular endothelial cells (PPMVECs), a marked degradation was observed. Post-infection, there was an absence of the surface fibrous glycocalyx [59]. Notably, the HP-PRRSV infection caused a profound alteration in the glycoprofiling of the membrane of PPMVECs.

Viral infections and their potential effects on the ECM represent an intriguing area of exploration in cellular biology. There is growing evidence to suggest that these infections influence the ECM, possibly disrupting its barrier function and even inducing inflammation in the tissue. Changes in the composition and organization of the ECM could, theoretically, promote viral invasion and replication. Yet, this relationship requires further examination to understand the full extent and implications of ECM remodeling due to viral interactions. In the realm of therapeutics, while certain antiviral drugs have been observed to mitigate inflammation and fibrosis in contexts like HCV [60,61,62], the specifics of their impact on the ECM remain less clear. This highlights our ever-evolving comprehension of the intricate interaction between viruses and the ECM, emphasizing the necessity for continued research.

### 2.2. Viral Manipulation and Interaction with Host Cell Membrane

Viral infections affect the membrane components of cells in various ways, depending on the specific virus and the host cell type [17,63]. Viruses interact with cell surface receptors, membrane proteins, and lipids to facilitate entry into the host cell and promote viral replication [28,64,65,66]. For example, the spike protein of SARS-CoV-2 facilitates virus entry into host cells by interacting with the ACE2 receptor on the cell membrane [67]. HIV binds to the CD4 receptor on T cells [68], while the influenza virus binds to sialic acid receptors on epithelial cells in the respiratory tract [69,70,71]. Additionally, some membrane-associated host factors, such as cholesterol and lipid rafts, play important roles in viral entry and replication [72,73,74]. Moreover, viral infections can disrupt membrane integrity and induce membrane damage [75], which can trigger immune responses and affect cell signaling and communication [76]. These interactions facilitate viral entry into the host cell, which can lead to viral replication and the spread of infection.

Viral infections impact other membrane components, such as lipids and cholesterol [77]. Many viruses require specific lipid- or cholesterol-rich environments for replication [74,78], and they can manipulate host cell membranes to create favorable conditions [79]. Viruses have been shown to induce lipid production, accumulating cholesterol and sphingoid bases in the host cell membrane [77,78]. HCV utilizes a strategy called membranous webs (MW) to alter the composition and function of host cell membranes to induce a membrane status conducive to virus replication [80]. HCV exploits these lipid components to optimize its replication, with the NS4B protein playing a critical role in forming a unique membranous web structure, essential for viral RNA replication [81]. NS5A, another viral protein, modulates host cell pathways, including those involved in lipid metabolism, leading to the formation of lipid droplets, further facilitating the HCV life cycle [82]. This interaction leads to a shift in the lipid composition of the host cell membranes, particularly in the formation of lipid droplets [78]. NS5B, the RNA-dependent RNA polymerase of HCV, not only plays a central role in the synthesis of the viral RNA genome but is also integral to the functional assembly of the membranous web. Its polymerase activity is critical in organizing the replication complex within these membrane alterations [83]. Moreover, the interaction of NS5B with other viral proteins and host cell components ensures the structural integrity and functionality of the membranous web, making it a hub for HCV replication [84]. Additionally, the interferon-stimulated gene SAMHD1 plays a role in restricting HCV replication by downregulating lipid synthesis [85]. These alterations in membrane lipid components emphasize the strategy of the virus to create an environment conducive to its lifecycle while highlighting the broader implications for the cellular health of the host. These complex interactions between viruses, the cellular vesicle machinery, and host immune defenses can ultimately lead to a variety of cellular changes and disease outcomes, so it is important to better understand the mechanisms underlying virus-induced impairment of vesicle formation and transport.

Viruses in the flavivirus family induce membrane remodeling to produce vesicles specialized for viral replication and assembly [86]. These vesicles not only provide a protective environment for viral replication but also facilitate the escape of the virus from the cellular immune response and improve the efficiency of virus transmission to other cells [87,88,89]. In addition, HIV can induce cell membrane remodeling through the formation of viral synapses that facilitate the cell-to-cell transmission of the virus [90,91]. These changes can disrupt cellular homeostasis and contribute to the pathogenesis of HIV. Human cytomegalovirus (HCMV) also remodels the plasma membrane, involving the modulation of cell surface receptors. During HCMV infection, the expression of these receptors undergoes changes, and this alteration is driven by interferon-dependent processes [92]. These modifications were discovered within macrophages, suggesting pathways for HCMV to evade the immune system. HCMV evades immune detection by altering membrane proteins and downregulating MHC class I molecules, impacting cell-to-cell interactions [93]. Research suggests that HCMV spreads within host cells by ‘hiding’ from immune responses, utilizing a strategy of cell-to-cell transmission within persistent sanctuary sites [94]. RSV significantly remodels the plasma membrane, facilitating its cell-to-cell transmission as well. The virus induces syncytium formation through its fusion protein, allowing direct spread between adjacent cells while evading extracellular immune defenses [95]. RSV also alters the plasma membrane for the selective budding of virions, an essential step for efficient neighboring cell infection. Additionally, the remodeling of the actin cytoskeleton, influenced by RSV, potentially enhances the spread of the virus by altering cell morphology and promoting closer cell contact [96,97]. The herpes simplex virus (HSV) also has the ability to remodel the plasma membrane of infected cells. During infection, HSV-1, in particular, alters cell membranes through the degradation of GOPC, a protein that leads to significant changes in the membrane structure [98]. In parallel with membrane remodeling, HSV employs cell-to-cell transmission as a means to spread. This allows the virus to move from one infected cell to adjacent uninfected cells, serving as a strategic evasion mechanism against host immune responses [99].

HIV infection profoundly impacts the cell surface protein composition of its primary target cells, particularly CD4+ T cells, employing sophisticated strategies to evade the immune defenses of host and enhance its replication. An innovative HIV reporter virus was used to conduct a detailed proteomic analysis of HIV-infected primary human CD4+ T cells, identifying approximately 650 HIV-dependent changes in protein expression, including novel Vif-dependent targets and proteins not regulated in T-cell lines. These findings underscore the unique interactions between HIV and primary cells, differing significantly from those in cell lines [100].

The manipulation of cell surface proteins by HIV extends to its evasion of the immune system. For instance, it was revealed how the HIV-1 Nef protein orchestrates the downregulation of CD4, a key receptor for the virus, through complex interactions with the cellular machinery of the host. This mechanism not only aids in the virus’s escape from immune surveillance but also facilitates its replication and spread within the host [101]. Such insights into the intricate interplay between HIV and the host cell surface proteins are crucial in understanding the virus’s pathogenicity and in developing targeted therapies to combat HIV infection. The research underscores the complexity of the life cycle of HIV and its ability to strategically modify and utilize host cell proteins to its advantage, a factor that poses significant challenges in the development of effective treatments and vaccines. These studies not only contribute to our fundamental understanding of HIV biology but also open up avenues for novel therapeutic strategies that could target these specific interactions between the virus and the host cell surface proteins.

Viral infections demonstrate a sophisticated ability to manipulate and interact with cellular membrane components, tailored to the specific virus and host cell involved. Through strategic engagements with receptors and lipids, viruses like SARS-CoV-2 and HIV optimize their entry and replication processes. The alterations in lipid and cholesterol dynamics, particularly evident in the case of HCV, not only facilitate the viral lifecycle. They also have broader implications for host cellular health and disease manifestation. The profound membrane alterations further put stress on the depth and complexity of these viral-host interactions.

### 2.3. Viral Infection Alters the Endocytic Pathways

The vesicular transport system plays an important role in inter- and intracellular communication [102,103]. Enveloped viruses utilize the membrane transport machinery of the host to enter the cell, transport their components, and use this system to exit the cell as newly synthesized virions [104,105,106]. Viruses not only use the transport system for the infection but also manipulate the vesicle transport systems to create an environment conducive to their proliferation and shedding from cells [79,107,108].

Endocytosis, a cellular mechanism that facilitates the internalization of extracellular molecules, is a prime target for viral proliferation. Viruses can induce the formation of vesicles that internalize them into the host cell. For instance, viruses can engage receptors on the cell surface that activate clathrin-mediated or caveolae-mediated endocytosis, leading to their uptake within clathrin-coated or caveolin-rich vesicles, respectively. For viruses lacking specific receptor targets, they prompt the host cell to non-selectively draw in extracellular fluid through macropinocytosis, capturing the virus within large vesicles. The crucial step for all viruses following entry is to exit these vesicles, often facilitated by the acidic environment of endosomes, which prompts a release into the host cytoplasm, commencing the infectious cycle. Viruses manipulate endocytic pathways to ensure their successful entry, replication and spread [109]. This relationship between viral infection and endocytosis is mediated by specific host genes and proteins, often subverted by viruses to further their lifecycle.

As identified through a functional genomic screen, HIV targets pivotal components of the endocytic machinery [110]. The Vpu protein of HIV-1 disrupts the cellular endocytic process by preventing the endocytosis of nascent retrovirus particles, promoting their release from the plasma membrane instead [111]. Another intricate aspect of this manipulation involves the Tat of HIV-1, which directly influences the cytoskeleton and vesicular traffic of the host, implying subtle controls exerted over endocytic routes [112].

The African swine fever virus (ASFV) encodes over 150 proteins, many of which have unknown functions. Recent proteomic analyses have identified potential interacting partners for ASFV proteins like P34, E199L, MGF360-15R, and E248R [113]. These proteins are associated with pathways involving intracellular and Golgi vesicle transport, endoplasmic reticulum organization, lipid biosynthesis, and cholesterol metabolism. Notably, Rab proteins, essential regulators of the endocytic pathway, have been identified as interactors of both P34 and E199L. These proteins coordinate a tight regulation of the endocytic pathway, crucial for ASFV infection. Influenza viruses are related to Rab proteins as well. The viruses affect the movement of Rab11A, a component integral to the intracellular transport of viral RNA, through reduced dynein association, indicating a strategic alteration of the endocytic pathways to favor the viral lifecycle [114].

Epstein–Barr virus (EBV), which causes mononucleosis and is associated with several cancers, encodes a protein called LMP2A [115]. This protein alters the endocytic pathway by interacting with host cell signaling molecules. LMP2A disrupts the normal endocytic cycling of the B-cell receptor (BCR), leading to its continuous internalization and degradation. This manipulation dampens BCR signaling, allowing the virus to evade the immune response and establish a persistent infection.

By altering specific proteins and pathways, viruses ensure their survival and replication within host cells. Understanding these specific manipulations is crucial for gaining insights into the fundamental viral infection mechanism.

### 2.4. Virus Remodels the Cytoskeleton

Recent research has elucidated the diverse mechanisms by which viruses manipulate the host cell cytoskeleton to facilitate their lifecycle and pathogenesis. For instance, the baculovirus Autographa californica multiple nucleopolyhedrovirus (AcMNPV) regulates the host actin cytoskeleton through its actin rearrangement-inducing factor 1 (Arif-1), leading to pronounced F-actin remodeling in mammalian cells, a phenomenon observed across various cell types [116]. Similarly, human-tropic alphaherpesviruses, such as HSV and varicella-zoster virus, target cytoskeletal elements like actin filaments and microtubules, crucial for viral pathogenesis [117]. In the realm of HIV-1, the interaction with CD4+ T cells involves the cytoskeleton significantly, with the actin and tubulin cytoskeleton directing crucial processes like assembly, budding, and the formation of a virological synapse [118]. Furthermore, the mammalian Diaphanous-related formin-1 (mDia1) has been shown to control cytoskeleton dynamics during human influenza A virus infection, playing a role in restricting infection initiation [119]. Studies on Herpes simplex virus 1 and rotavirus have also underscored the importance of cytoskeleton remodeling in viral replication and exit strategies [120,121]. Additionally, the HTLV-1 Tax protein and HIV-1 exposure have been found to significantly affect cytoskeletal dynamics, with the former facilitating viral transmission through cytoskeleton remodeling [122] and the latter inducing PKG1-mediated phosphorylation and the degradation of stathmin, leading to compromised epithelial barrier integrity [123,124]. These studies collectively highlight the pivotal role of cytoskeleton in viral infection and pathogenesis.

### 2.5. Virus and Nuclear Remodeling

Viruses, both DNA and RNA types, often rely on host nuclear proteins for their replication. To access the host cell’s nuclear proteins for their replication, viruses remodel the nucleus by employing various strategies.

In the case of HIV, key proteins in the pre-integration complex contain nuclear localization signals (NLS) in their genome sequences. These NLS facilitate interaction with nuclear transport receptors, enabling the transport of viral proteins through the nuclear pore complex (NPC) [125]. Similarly, the influenza virus nucleoprotein (NP) possesses NLS1, NLS2, and NLS3, and these signals enable NP to bind with cellular importins, facilitating the transportation of the viral ribonucleoprotein complex (vRNP) into the host cell nucleus [126]. Viruses such as HSV-1, adenovirus, and hepatitis B virus (HBV) interact with the host nuclear pore complex (NPC) via their capsids, either directly or assisted by importins [127,128,129]. This interaction serves as a signal for capsid disassembly, allowing the viral genome and associated proteins to enter the nucleus through the NPC. The disruption of the nuclear envelope (NE) and nuclear lamina is observed in parvovirus infection and the disruption is considered to facilitate viral entry [130].

Upon successful entry into the nucleus, both DNA and RNA viruses exploit various nuclear components to facilitate their replication. For instance, viruses such as HSV and adenovirus re-localize or disrupt host nucleolar proteins [131,132]. In addition, the nucleolar localization of viral capsids and RNA-binding proteins is a common phenomenon in many RNA viruses [133]. Viruses such as HIV-1, poliovirus, and encephalomyocarditis virus (EMCV) localize their proteins in the nucleolus, and this leads to the downregulation of the host system and the facilitation of viral assembly at the same time [134,135,136]. Some viruses, including HSV-1, have been reported to induce the redistribution and degradation of host promyelocytic leukemia nuclear bodies (PML NBs). These PML NBs, which have the ability to be SUMOylated, act as a target for viruses to escape the antiviral signaling response in order to neutralize the antiviral response [137,138,139]. During viral infections, several other subnuclear structures, including Cajal bodies [140], the nuclear lamina [141], as well as chromatin [142], are also targeted.

### 2.6. Virus and Modification of Endoplasmic Reticulum

The endoplasmic reticulum (ER) is a continuous membrane system with specialized subdomains that occupies a large part of the cytoplasm of eukaryotic cells. The ER not only serves multiple functions, including protein synthesis, modification, transport, and calcium homeostasis, but also plays an important role in lipid synthesis [143,144,145,146]. Almost all eukaryotic cells possess the ER. In animal cells, the ER generally accounts for over half of the membranous content within the cell.

In the interplay between viruses and host cells, the ER plays a pivotal role. In particular, positive-strand RNA viruses, in which genome replication occurs in the association with virus-induced membranes, are known to actively exploit host membrane structures including the ER (Figure 2B) [147,148]. During infection with positive-strand RNA virus, the ER’s structure is often dramatically changed due to the active interaction between viral membrane proteins and host membrane proteins. This interaction contributes to forming a replication organelle (RO) with unique lipid composition, facilitating robust virus replication [149,150,151]. The RO is known to be a structure that concentrates viral proteins and host proteins into the proper spot, mediating the proper topology of the replication machinery [152]. In addition, the RO may be involved in the protection of viral RNA from RNA degradation, which is mediated by the host cellular RNase [153].

Membrane sources for RO are different membranous organelles such as ER, golgi apparatus, peroxisomes, mitochondria, and plasma membrane. Among the membrane-bounded organelles, the ER represents the main membrane source for many positive-strand RNA virus ROs in animal cells [154]. The majority of ROs are vesicular structures, and single membrane vesicles (SMVs) are the simplest form, which are typically 50–200 nm in diameter [155,156]. Some SMVs, called spherules, have small pores inside, which link either vesicles to each other or the vesicle lumen to the external environment [157]. Multiple SMVs can be packed together in an organelle to form the higher-order vesicle packets (VPs) [147]. RO structures known as double-membrane vesicles (DMVs) have more intricate structures with 200–400 nm diameter [158]. More complex RO organizations such as multi-vesicular bodies (MVBs), multi-membrane vesicles (MMVs), or convoluted membranes (CMs) also occur [150,159,160]. MVB structures consist of big vacuoles containing numerous small disordered membranous vesicles. MMVs consist of big multi-layered membranous particles, tubule-like structures, and/or the zippered ER. CMs consist of massive unstructured membranous aggregates with or without vesicles.

Viruses, including brome mosaic virus (BMV) and coronavirus, induce CM formation either in the perinuclear region or randomly dispersed locations in the cytoplasm [150,161]. Other viruses such as beet black scorch virus (BBSV) or tobacco necrosis virus (TNV-W) induce ER membrane dilations and invaginations that are rounded structures [162]. Viruses also form higher-order vesicle packets (VPs) with small vesicles 50–100 nm in size, and these vesicular structures are the areas where viruses replicate their genome [147]. The spherule structures in BBSV-infected cells and flaviviridae virus family-infected cells have a spherule vesicle with a narrow neck (5–10 nm in diameter) linked to the VP membrane and thus connecting the spherule interior to the cytoplasm [162,163,164]. In contrast to BBSV or the flaviviridae family, the ROs of peanut clump virus (PCV) in tobacco protoplasts form different type of MVBs [165]. These MVBs contain multiple disordered membranous vesicles often in one row of vesicles and surrounded by a single membrane. Interestingly, turnip mosaic viruses (TuMV) do not only induce the formation of SMVs but also of DMV–like structures that are found in the perinuclear cytoplasmic region [166]. The DMVs formed during TuMV infection occur concomitantly during the late stage of infection with massive membrane rearrangements leading to altered endomembrane structures such as a dilated ER and the membranous inclusion bodies.

Recent studies have reported the interactions between viral infection and host cellular ER-shaping proteins such as ATL3, RTN3, and Lunapark, which are involved in ER network formation [167]. ER-shaping atlastin proteins (ATL1, -2, and -3), which induce ER membrane fusion, are recruited to the Zika virus (ZIKV) replication site and co-localized with the viral proteins, facilitating ZIKV replication [168]. ATL3s can also regulate Kaposi’s sarcoma-associated herpesvirus (KSHV) lytic activation and infection [169]. RTN3, which stabilizes ER membrane curvature, is known to have a relationship with the replication of viruses including DENV, WNV, ZIKV and SARS-CoV-2 [86,170]. Lunapark, an ER membrane protein that typically stabilizes three-way ER junctions, has been suggested to have pivotal roles in viral infection. This is achieved by relocating to the ER foci, where it supports viral ER-to-cytosol escape [171]. Even though the function of membranous structures observed during viral infection has been poorly investigated, in some instances, ROs have direct contact sites with viral particles. It can be inferred that these structures are either correlated with viral replication or represent abnormal cellular structures arising due to altered membrane metabolism, or a feature of the cellular process to limit the viral infection. Furthermore, numerous alterations in organelles observed during viral infections are recognized as being reminiscent of processes regulated by membrane contact sites (MCSs) [172]. ER is considered as a “master regulator” of MCS biology by directly connecting every other organelle in the cell [173]. Researchers have suggested that all organelles can communicate through MCSs, and the dysregulation of organelle MCSs is closely associated with diseases such as neurodegeneration and cancer [174]. Therefore, the exploitation of ER during viral infections leading to alterations in MCSs may be a crucial factor in the development of viral infection-related diseases, including cancer [175]. Therefore, understanding about ROs, which may be mainly derived from ER, would be important in virus studies.

### 2.7. Alternation to Mitochondrial Morphology and Function during Viral Infection

Mitochondria are known to be dynamic organelles repeating frequent cycles of fusion and fission, which means the joining of mitochondria and fragmentation of mitochondria, respectively. Fusion and fission are important in mitochondria-associated functions and homeostasis [176,177]. Mitofusin1 (MFN1) and MFN2, located in the outer mitochondrial membrane, are involved in mitochondrial fusion by mediating the fusion of adjacent mitochondria [178]. Optic atrophy 1 (OPA1), located in the inner mitochondrial membrane, not only plays a crucial role in maintaining the integrity of the cristae but also in mitochondria fusion [179,180,181]. On the other hand, in mitochondrial fission, dynamin-related protein 1 (DRP1) is a key protein, and some DRP1-interacting proteins, such as fission 1 (FIS1) and mitochondrial fission factor (MFF), are important [182,183,184]. Mitochondrial turnover is another important factor in mitochondrial dynamics, and mitochondrial turnover is closely linked to mitochondrial fission. Mitochondrial turnover is regulated by a process termed mitophagy, an autophagic system in mitochondria [185].

Regulating mitochondrial dynamics is crucial not only for maintaining host homeostasis and a robust defense system but also for influencing viral infection (Figure 2C) [186,187]. Hence, host effectors and viral factors actively compete to control mitochondrial morphology. For instance, hepatitis C virus (HCV) and hepatitis B virus (HBV) promote mitochondrial fission by regulating phosphorylation of DRP1 at S616, resulting in DRP1 recruitment to mitochondria [188,189]. HCV-induced fission is followed by mitophagy that attenuates apoptosis, and these alterations are associated with enhanced viral secretion and suppressed IFN synthesis [189]. On the other hand, mitochondrial fusion is antagonized via the cleavage of MFN1 and MFN2 by DENV viral protease, leading to IFN-I RLR signaling impairment [190].

The clearance of mitochondria via mitophagy is another key factor both for the host defense system and viral infection [186,191]. During mitophagy, damaged mitochondria that are impossible to be repaired are recycled be being “tagged” with autophagosomal markers to form autophagosomes, which fuse with a lysosome in the end [192]. Viral induction of mitophagy is common in infected cells [193,194]. Viruses such as HCV and HBV trigger mitophagy by inducing the translocation of Parkin to mitochondria [188,195]. Mitophagy in these infected cells attenuates apoptosis and thus allows for persistent viral infection. The direct binding of viral proteins to host mitophagy effectors resulting in the counteraction of antiviral responses has been demonstrated. The matrix protein (M) of human parainfluenza virus type 3 (HPIV3) binds to the Tu translation elongation factor mitochondrial (TUFM), a receptor for mitophagy initiation. This binding in turn leads to the induction of Parkin-independent mitophagy [196]. M-induced mitophagy leads to the inhibition of the IFN-I response.

Not only mitochondrial morphology but also mitochondrial functions are affected by viral infection. For instance, the TCA cycle has been suggested to be a target of some virus species [197,198]. The reason why viruses rewire the TCA cycle is poorly investigated currently, but there is a probable hypothesis that may explain the reason for this, namely that the fatty acid (FA) synthesis pathway produces fatty acids derived from acetyl-CoA as well as other TCA intermediates. This rewiring aims to increase lipid synthesis, including long-chain fatty acids required to produce membranous structures for virions [199,200]. From a similar perspective, the fact that viruses can also perturb fatty acid oxidation, which occurs in the mitochondrial matrix, can be interpreted in the same way. Researchers have suggested that viruses may hijack fatty acid oxidation to facilitate viral growth. However, the underlying mechanisms by which viruses alter fatty acid oxidation to subvert host defenses have not been fully investigated [201,202].

The morphological and functional changes of mitochondria during viral infection can induce antiviral responses. Mitochondrial antiviral signaling (MAVS) is a protein located on the outer mitochondrial membrane that plays a crucial role in the induction of the host’s innate immune response to viral infections [203]. So, shifted mitochondrial dynamics caused by viral infection can inhibit MAVS [204]. Notably, specific subdomains of mitochondrial-ER junctions, termed mitochondria-associated membrane (MAM), are crucial for MAVS signaling induction, and the disruption of MAMs by viral infection leads to a compromised antiviral immune response [205]. Furthermore, metabolic changes during viral infection also contribute to antiviral responses. Glycolytic metabolites exert a negative regulatory effect on antiviral signaling, including MAVS, while simultaneously facilitating viral nucleotide synthesis [206,207].

### 2.8. Cellular Condensates and Viral Replication

Cells contain specialized compartments consisting of membrane-bound and membraneless organelles that organize physiological processes. However, the function of the membraneless organelles has not been fully investigated compared to that of membrane-bound organelles such as ER, Golgi, and the mitochondria. Since physical mechanisms and molecular interactions that govern membraneless organelles have been revealed, the new renaissance that deals with cellular functions from the perspective of the function of membraneless organelles is emerging [208]. The membraneless organelles, now referred to as biomolecular condensates, include nucleoli, Cajal bodies, P-bodies, stress granules, processing bodies, and signaling compartments [209]. In most cases, biomolecular condensates form through a process called ‘phase separation’, in which the cytoplasm or nucleoplasm separates into two or more membraneless compartments [209]. Emerging evidence indicates that interactions between RNA and proteins containing IDR region are one of the major components inducing the formation of biomolecular condensates [210]. Although the formation of biomolecular condensates, which is thought to mediate viral replication and nucleocapsid assembly, has long been recognized, there is a chicken and egg situation in this phenomenon. However, accumulating evidence suggests that viruses are truly masters of phase separation and induce biomolecular condensate formation. [211]. Based on this concept, the RNA virus proteome, enriched in IDR-containing proteins, contributes to phase separation and induces biomolecular condensates. These condensates, in turn, contribute to viral replication and nucleocapsid assembly [212,213]. In particular, two types of viral protein, namely antiterminators and nucleocapsid proteins, have features and roles consistent with their ability to phase separate with viral RNA molecules [212,214].

As such, why some viruses promote or inhibit formation of biomolecular condensates is well-characterized, and researchers have suggested that dynamism (assembly/disassembly) of the biomolecular condensates such as P-bodies and stress granules during viral infection would allow the chronicity of the infection [215]. Although there are many hurdles for developing drugs targeting biomolecular condensates, the shared mechanisms employed by various viruses to modulate these condensates are notable. This suggests that drugs targeting biomolecular condensates could eventually serve as broad-spectrum antiviral reagents [216].

Compared to the positive-sense RNA viruses, both replication organelles and nucleocapsid assembly sites are intimately associated with modified cellular membranes. However, a different scenario is observed in negative-sense RNA (as well as dsRNA) viruses. In these viruses, the specialized compartments are typically cytoplasmic, membraneless organelles, commonly referred to as inclusion bodies [211,217]. In many cases, inclusion bodies are used as histological proof of infection, and recent studies have suggested that viral RNA synthesis, as well as nucleocapsid assembly, occurs at these sites [218,219,220]. As such, inclusion bodies can be regarded as viral replication organelles or factories. For both negative- and positive-sense RNA viruses, accumulating evidence suggests that replication organelles and nucleocapsid assembly sites are formed via phase separation, mediated by viral antiterminator or nucleocapsid proteins [212,214]. It can be observed that these characteristics manifest as structural changes in the ER in positive-sense RNA viruses. In negative-sense RNA viruses, they appear to be expressed as cellular condensate formation.

### 2.9. Viral Manipulation of Autophagosomes and Viral Replication

The relationship between viruses and autophagy has long been investigated and many researchers have compared the relationship between viruses and autophagy to a double-edged sword [221]. In general, autophagy is considered to be one of the most powerful tools for host cells to fight against viral infection. However, viruses can exploit autophagy for their survival as well. In particular, accumulating evidence has supported that RNA viruses manipulate autophagy for their replication [222,223,224]. During RNA virus infection, double-membrane compartments form upon the activation of autophagy (Figure 2D). These compartments provide a physical platform for viral replication and a place for the local concentration of essential intermediates for the virus. Additionally, they offer a shelter for the virus to avoid detection by innate immune sensors and degradation machineries [225,226].

Poliovirus, a serotype of the species Enterovirus C in the Picornaviridae family, is known to form DMVs in the cell that resemble autophagosomes and provide scaffolds for viral RNA replication [227]. In addition, poliovirus infection led to the accumulation of LC3 in puncta. The expression of the viral proteins 2BC and 3A led to LC3 lipidation and DMV formation, which provides a mechanistic link between autophagy and poliovirus replication [228]. Other picornaviruses, such as Coxsackievirus B3 (CVB3) and foot-and-mouth disease virus (FMDV), also use autophagy for replication. These viruses induce autophagosome formation during their infections, and the autophagosomes contribute on their replication [229,230]. However, the underlying mechanisms of how different types of picornaviruses modulate autophagy are different [231,232]. Coronaviruses, such as SARS-CoV, SARS-CoV-2 and mouse hepatitis virus (MHV), also induce the formation of DMVs and use autophagy for their replication [148,158,226]. In particular, in the case of SARS-CoV-2, autophagy is largely targeted by viral proteins, including E, M, ORF3a, ORF7a, and Nsp15. This results in the decrease in autophagic degradation and the accumulation of autophagosomes, which can be exploited by SARS-CoV-2 for its replication, maturation, and egress [50,233]. Flaviviruses such as DENV and West Nile virus (WNV) have structural proteins that can induce autophagy in an unfolded protein response (UPR)-independent manner [234,235]. In addition, in line with the fact that autophagy and ER processes are frequently connected, active ER rearrangement is induced by flaviviruses via ER-shaping proteins. This contributes to the formation of autophagy-like structures that serve as sites of viral RNA replication and virion assembly [147,236,237,238]. Viruses, including HCV human parainfluenza virus type 3 (HPIV3), induce autophagosome accumulation by blocking autophagosome–lysosome fusion. However, the underlying mechanisms vary according to the viruses [239,240]. Autophagy activated by viruses has been suggested to be utilized for viral exocytosis as well [230,241,242].

### 2.10. Viral Exocytosis and Lysosome

A lysosome is a spherical vesicle-shaped membrane-bound organelle containing more than 60 different hydrolytic enzymes that can break down a variety of biomolecules. The balance between protein synthesis and degradation is important for protein homeostasis, also known as proteostasis, and maintaining protein homeostasis is critical for cell survival [243]. In eukaryotic cells, protein degradation is regulated by proteasomes and lysosomes. Proteasomes, including ER-associated degradation (ERAD), generally eliminate short-lived proteins and soluble misfolded proteins via ubiquitination [244,245], whereas lysosomal degradation, including the autophagic- and endo-lysosomal system and ER-to-Lysosome associated degradation (ERLAD), is responsible for the degradation of long-lived proteins and insoluble protein aggregates. It also degrades entire organelles, macromolecular compounds, and intracellular parasites via endocytosis, phagocytosis, or autophagy pathways [246,247,248]. Beyond the consolidated role in degradation and recycling, lysosomal degradation systems have been reported to play a crucial role in extracellular release pathways. This role is not only important for cell fitness but also for cell-to-cell communication [249].

In line with the close relationship between viral infection and autophagy, lysosomes are thought to play a crucial role in viral infection because lysosome is a bona fide component in autophagic degradation. Very recent papers have provided reports about the mechanisms of how viruses, such as β-coronaviruses, reoviruses, and arboviruses, utilize the lysosome for their viral exocytosis instead of the biosynthetic secretory pathway [34,250,251]. All of these viruses achieve the non-lytic release of their viral particles by inhibiting acidic pH gradients inside of the lysosome, which is generated and maintained by using the activity of a proton-pumping V-type ATPase, resulting in impeding the activation of lysosomal enzymes [252]. In particular, the underlying mechanism of how the β-coronaviruses cope with the lysosome is widely investigated, and open reading frame protein 3A (ORF3a) of SARS-CoV-1 and SARS-CoV-2 has been reported to be important in lysosomal exocytosis-mediated viral egress of the viruses. According to various studies, ORF3a disrupts lysosomal acidification and SARS-CoV-2 ORF3a also promotes lysosomal exocytosis-mediated viral egress by regulating the BORC–ARL8b complex, which drives the anterograde transport of lysosomes from perinuclear regions to the vicinity of the PM and exocytosis-related SNARE proteins [253,254]. As a result, viral proteins within the lysosome can be transported outside of the cells instead of being degraded. This mechanism allows viruses to utilize deacidified lysosomes for their egress.

### 2.11. Viral Manipulation of Extracellular Vesicles

Extracellular vesicles (EVs) are diminutive membrane-bound structures released by cells into the surrounding extracellular matrix [255]. These vesicles play a crucial role in cellular communication, facilitating the transfer of proteins, lipids, viral components, and virions among cells [256]. The exploitation of EVs by viruses enables these pathogens to manipulate the EV pathway, thereby augmenting their infectivity, dissemination, and ability to evade the immune system. The understanding of this mechanism has opened up new avenues in comprehending viral pathogenesis and devising innovative diagnostic and therapeutic approaches.

The biogenetic pathway of EVs significantly overlaps with viral processes. For instance, the endosomal sorting complex required for transport (ESCRT) machinery and the RAB family of small GTPases, integral to EV formation, are also implicated in viral release. [257]. Specifically, herpes simplex virus 1 (HSV-1) harnesses the ESCRT complex to escape the nucleus, recruiting the ESCRT machinery and VPS4 for budding through the nuclear membrane [258,259]. Similarly, the hepatitis b Virus (HBV) employs the trafficking pathway of the RAB family. The large hepatitis B surface proteins (LHBs) initiate the translocation of HBV from the endoplasmic reticulum (ER) to multivesicular bodies (MVBs) following interaction with Rab5B [260]. Within MVBs, HBV commandeers Rab7a and Rab27, facilitating its egress outside the plasma membrane [261].

EVs have garnered significant attention for their role in intercellular communication, positioning them as promising candidates for therapeutic vehicles. Their ability to effectively deliver biomolecules between cells has catalyzed a swift advancement in the development of potential therapies that harness the unique capabilities of EVs [260]. In the context of viral infections, EVs can carry viral proteins from infected cells and have been implicated in the propagation of infections. This has spurred interest in utilizing EVs for vaccination applications and in the development of EV-based delivery carriers [261]. Additionally, EVs have emerged as competitors against viruses in the gene therapy. While viruses are traditionally engineered as gene vectors, EVs present numerous advantages, one of which is their customizable size. Furthermore, EVs offer versatility, allowing for optimization in diverse contexts and for a range of applications, thus broadening their potential in gene therapy strategies. While EVs can be innovative therapeutics, they have limitations. One key challenge is the purification of EVs due to the similarity in size between EVs and viruses. This similarity complicates the isolation of EVs, as there is a risk of viral contamination. However, if this hurdle can be effectively solved, EVs could be utilized in the development of vaccines and as highly effective tools for transporting various biological materials.

## 3. Conclusions

Viruses, despite their relative simplicity, have evolved to exploit the host cell machinery with remarkable precision and efficiency. This manipulation is evident in various aspects of host cell biology, ranging from alterations in the extracellular matrix and cell membrane to the intricate modulation of endocytic pathways, the endoplasmic reticulum, the mitochondria, and other subcellular organelles (Table 1).

In conclusion, this review presents a comprehensive overview of the numerous ways in which viruses manipulate host cellular processes and structures. The intricate dance between viruses and host cellular processes, from the ECM to the manipulation of organelles, emphasizes the adaptability and resilience of viruses. Understanding these interactions is pivotal for advancing antiviral strategies and gaining deeper insights into cellular biology. Understanding the adaptability and resilience of viruses will contribute to the development of effective antiviral strategies. Future research should continue to explore these interactions in greater depth, aiming to improve our global preparedness for viral pandemics.

## Figures and Tables

**Table 1:** Viral Manipulations of Host Subcellular Organelles.

Host Subcellular Organelles	Highlights	Viruses	References
ECM	ECM alternation resulting in activation of inflammatory mediators	HCV, CHIKV	[42,43,44,45,46,47,48,49,50,51,52,53]
	HA-enriched ECM	RSV	[54,55,56,57,58]
	Alteration in the glycoprofiling	HP-PRRSV	[59]
Cell Membrane	Lipid and cholesterol manipulation	HCV	[78,79,80,81,82,83,84,85]
	Membrane remodeling	HIV, HCMV, etc.	[63,88,89,90,91,92,93,94,95,96,97,98]
	Manipulation of cell surface protein	HIV	[99,100]
Cytoskeleton	Control cytoskeleton dynamics	AcMNPV, HSV, etc.	[115,116,117,118,119,120,121,122,123]
Nucleus	Remodel nucleus for viral entry	HIV, HSV-1, etc.	[124,125,126,127,128,129]
	Exploit nuclear components for viral replication	HSV, HIV-1, etc.	[130,131,132,133,134,135,136,137,138]
Endoplasmic Reticulum	Remodel ER membrane to form replication organelles	BMV, BBSV, etc.	[149,160,161,162,163,164,165]
	Exploit ER-shaping proteins	ZIKV, KSHV, etc.	[86,166,167,168,169]
Mitochondria	Regulate mitochondrial fusion and fission	HCV, HBV, etc.	[187,188,189]
	Induction of mitophagy	HCV, HBV, etc.	[185,187,190,191,192,193,194,195]
	TCA cycle regulation	HIV, IV, etc.	[196,197,198,199,200,201]
Cellular condensates	Promote or inhibit formation of biomolecular condensates	SARS-CoV-2, HIV-1, etc.	[210,211,212,213,214,215,216]
Autophagosome	Induce autophagosome or autophagy-like structure formation	SARS-CoV-2, MHV, etc.	[50,146,232,233,234,235,236,237,238]
	Autophagosome accumulation by blocking autophagosome–lysosome fusion	HCV, HPIV3	[238,239]
Lysosome	Utilize the lysosome for viral exocytosis	SARS-CoV-1, SARS-CoV-2	[249,250,251,252]
Extracellular Vesicles	Exploit ESCRT machinery or RAB family	HSV-1, HBV	[256,257,258,259]

## Data Availability

Not applicable.
